# Causal Relationship Analysis of the Patient Safety Culture Based on Safety Attitudes Questionnaire in Taiwan

**DOI:** 10.1155/2018/4268781

**Published:** 2018-03-01

**Authors:** Yii-Ching Lee, Pei-Shan Zeng, Chih-Hsuan Huang, Hsin-Hung Wu

**Affiliations:** ^1^Quality Management Center, Shanghai Changtai Medical Technology Co. Ltd., Shanghai, China; ^2^The School of Health Policy and Management, Chung Shan Medical University, Taichung City, Taiwan; ^3^Department of Health Business Administration, Hung Kuang University, Taichung City, Taiwan; ^4^Department of Business Administration, National Changhua University of Education, Changhua, Taiwan; ^5^Qisda Corporation, Taoyuan City, Taiwan; ^6^School of Business Administration, Hubei University of Economics, Wuhan City, Hubei Province, China; ^7^Institute for Development of Cross-Strait Small and Medium Enterprise, Hubei University of Economics, Wuhan City, Hubei Province, China; ^8^Department of M-Commerce and Multimedia Applications, Asia University, Taichung City, Taiwan

## Abstract

This study uses the decision-making trial and evaluation laboratory method to identify critical dimensions of the safety attitudes questionnaire in Taiwan in order to improve the patient safety culture from experts' viewpoints. Teamwork climate, stress recognition, and perceptions of management are three causal dimensions, while safety climate, job satisfaction, and working conditions are receiving dimensions. In practice, improvements on effect-based dimensions might receive little effects when a great amount of efforts have been invested. In contrast, improving a causal dimension not only improves itself but also results in better performance of other dimension(s) directly affected by this particular dimension. Teamwork climate and perceptions of management are found to be the most critical dimensions because they are both causal dimensions and have significant influences on four dimensions apiece. It is worth to note that job satisfaction is the only dimension affected by the other dimensions. In order to effectively enhance the patient safety culture for healthcare organizations, teamwork climate, and perceptions of management should be closely monitored.

## 1. Introduction

Shieh et al. [1] pointed out that studying causal relationships among critical factors enables the decision maker to understand the underlying principles of the relationship and then make the accurate predictions of future outcomes. Lee et al. [2] also depicted that identifying causal relationships among critical factors is essential in a healthcare organization in order to enhance the patient safety culture relentlessly. In recent years, patient safety has become a critical issue in healthcare organizations. Establishing a better attitude toward patient safety would result in the lower number of medical errors and the improvement of the patient safety culture in healthcare organizations [3]. To assess the attitude toward patient safety, the safety attitudes questionnaire (SAQ) developed by Sexton et al. [4] with six dimensions has been widely used worldwide to evaluate the patient safety culture of healthcare organizations from a medical staff's viewpoints.

In order to help hospital management enhance the patient safety culture continuously, it is critically important to know the relationships of six dimensions from the SAQ and how each dimension interacts with others. Through examining the cause-effect relationship, the decision maker can initiate any improvement through causal dimension(s) that would have direct and significant influences on effect-based dimensions [5]. On the other hand, if the decision maker emphasizes the enhancement on effect-based dimension(s), the improvement might be limited due to complicated causal relationships among dimensions. The decision-making trial and evaluation laboratory (DEMATEL) method is one of the effective methods commonly used in practice to construct causal relationships among factors by a group of experts [1]. In addition, the DEMATEL method is intended to identify the interdependence among the elements of a system through a causal diagram to depict the basic concept of contextual relationships and the strengths of influence among the elements by a hierarchical structure [6, 7].

Lee et al. [2] applied the DEMATEL method to examine the contextual relationships among six dimensions of the SAQ to help the decision maker in healthcare organizations to take improvement actions more effectively from causal viewpoints. However, as time goes by, the relative causal relationships might be changed due to different perceptions and understanding of the patient safety culture perceived by experts. Therefore, this study intends to assess the contextual relationships of the six dimensions of the patient safety culture and then further make a comparison between the results in terms of similarities and differences. In doing so, hospital management can update the causal relationships among six dimensions in order to continuously improve the patient safety culture in Taiwan.

This paper is organized as follows.  Section 2 briefly summarizes the patient safety culture and safety attitudes questionnaire along with the DEMATEL method. The research method is depicted in Section 3. Results are summarized in Section 4. Finally, conclusions are provided in Section 5.

## 2. Literature Review

### 2.1. Patient Safety Culture and Safety Attitudes Questionnaire

Patient safety culture plays an important role to continuously improve patient safety in healthcare organizations [8]. A healthcare organization with a better patient safety culture can reduce the risk of patient safety issues [9]. Besides, a healthcare organization with a more open culture and reflective attitude toward errors and patient safety would reduce the number of accidents and failures [10]. Furthermore, regularly evaluating the patient safety culture helps hospital management monitor the changes and trends in a healthcare organization to identify weaknesses [11].

The safety attitudes questionnaire developed by Sexton et al. [4] has six dimensions including teamwork climate, safety climate, perceptions of management, job satisfaction, working conditions, and stress recognition. Teamwork climate is the perceived quality of collaboration between personnel. Safety climate is defined as the perceptions of a strong and proactive organizational commitment to safety. Perceptions of management are the approval of managerial action. Job satisfaction is the positivity about the work experience. Working conditions are the perceived quality of the work environment and logistical support. Finally, stress recognition is the acknowledgement of how performance is influenced by stressors [4].

Two previous researches have been found to study the causal relationships among dimensions based on the SAQ. Lee et al. [2] identified that teamwork climate, job satisfaction, perceptions of management, and working conditions are net causes, while safety climate and stress recognition are net effects. From an overall evaluation, teamwork climate is the most essential dimension for hospital management to improve the patient safety culture followed by perceptions of management. Lee et al. [12] evaluated the contextual relationships of nine dimensions from the Chinese version of the SAQ. Teamwork climate, job satisfaction, working conditions, hospital management support for patient safety, and teamwork across hospital units are causal dimensions, while safety climate, stress recognition, perceptions of management, and hospital handoffs and transitions are net effects. In summary, teamwork climate and hospital management support for patient safety are the two critical dimensions to improve the patient safety culture because these two dimensions have direct impacts on six dimensions except for stress recognition.

### 2.2. DEMATEL Method

Decision-making trial and evaluation laboratory method was originally developed by the Science and Human Affairs Program of the Battelle Memorial Institute of Geneva between 1972 and 1976 and was intended to study and solve the complicated and intertwined problems by improving the understanding through hierarchical structures [5]. This method is based on graph theory to solve the problems visually such that multiple criteria can be categorized into cause and effect groups in order to better understand the causal relationship [1]. The DEMATEL method uses arithmetic means to aggregate opinions from a group of experts [7]. The number of experts might vary from 7 to 21 depending upon the availability of experts [6, 13–15]. Therefore, there is no limit particularly the lower limit in the number of experts in the decision-making process when the DEMATEL method is applied [5]. In practice, the number of experts is dependent upon the availability of experts.

The DEMATEL method has been widely applied in healthcare management areas. For instance, Shieh et al. [13] identified a trusted medical staff with professional competence which is the most essential criterion that can have significant impacts on patient satisfaction. Nasiripour et al. [16] found the most important factor on the performance of prehospital emergency system in Iran. Mamikhani et al. [17] found critical factors affecting the compensation for services provided by emergency department nurses. In addition, Sener and Dursun [18] combined the fuzzy theory and DEMATEL method for supplier selections in healthcare industry. Further, Shieh et al. [19] used a modified DEMATEL method to set up a framework to evaluate the medical service quality by a case study. To sum up, the DEMATEL method can be applied in practice to evaluate causal relationships in healthcare industries.

Four major steps of the DEMATEL method are depicted below [15]:

Step 1: develop the average matrix. Each respondent uses an integer score of 0, 1, 2, and 3 representing the respective “no influence,” “low influence,” “medium influence,” and “high influence” to evaluate the direct influence between any two dimensions. The notation of *x*_*ij*_ is referred to as the degree to which the respondent believes dimension *i* affects dimension *j*. For *i* = *j*, the diagonal elements are set to zero, indicating no influence. An *n* × *n* nonnegative matrix is set up as **X**^*k*^ = [*x*_*ij*_^*k*^] for each respondent, where *k* is the number of respondents and *n* is the number of dimensions. If there are *H* respondents, the average matrix **A** = [*a*_*ij*_] is depicted below:
  (1) $$\begin{array}{c} a_{ij}=\frac{1}{H}\overset{h}{\underset{k=1}{\sum }}x_{ij}^{k}. \end{array}$$
Step 2: calculate the normalized initial direct-relation matrix **D** by the following equation, where each element in matrix **D** is between zero and one.
  (2) $$\begin{array}{c} D=A\times \frac{1}{\max_{1\leq i\leq n}\sum_{j=1}^{n}a_{ij}}. \end{array}$$
Step 3: compute the total relation matrix **T** by **T** = **D**(**I** − **D**)^−1^, where **I** is the identity matrix. Let *r* and *c* be *n* × 1 and 1 × *n* vectors representing the sum of rows and sum of columns from the total relation matrix **T**, respectively. The notation of *r*_*i*_ is to take into account both direct and indirect effects given by dimension *i* to the other dimensions by summing the values of the *i*th row in matrix **T**, whereas the notation of *c*_*j*_ is to take into account both direct and indirect effects by dimension *j* from the other dimensions by summing the values of the *j*th column in matrix **T**. When *j* = *i*, the sum (*r*_*i*_ + *c*_*j*_) is defined as the total effects given and received by dimension *i*, indicating the degree of importance for dimension *i* in the entire system. On the other hand, the difference (*r*_*i*_ − *c*_*j*_) is defined as the net effect that dimension *i* contributes to the system. Dimension *i* is a net cause when (*r*_*i*_ − *c*_*j*_) is greater than zero, whereas dimension *i* is a net receiver or result when (*r*_*i*_ − *c*_*j*_) is less than zero.
Step 4: Set up a threshold value for the digraph by computing the average of the elements in matrix **T**. The digraph can be plotted by mapping the dataset of (*r* + *c*, *r* − *c*).

Based on the current studies using the DEMATEL method, the opinions from experts are assumed to be valid without examining the consistency ratio [20–22]. Though Shieh and Wu [5] tried to evaluate the consistency ratio from the survey data, their research work was to identify those experts who have quite different viewpoints than the others. In reality, those experts might have unique viewpoints to be taken into consideration. In contrast, their opinions might be unreliable to be included in the further analyses. However, there is no standardized approach such as the consistency ratio used by analytic hierarchy process to assess the consistency opinions for the DEMATEL method. Therefore, the drawback or limitation of the DEMATEL method is that there is a lack of established tools or methods to assess the consistency ratio when the survey results are from the DEMATEL-based questionnaire.

## 3. Research Method

The designed questionnaire in this study shown in Table 1 is to assess the influence among six dimensions of the safety attitudes questionnaire, where 0, 1, 2, and 3 represent no influence, low influence, medium influence, and high influence, respectively, for each pair of six dimensions. Thirteen experts in the patient safety culture, medical quality, or human resource management including physicians, nurses, administrators, and professors with at least five years of working experience were invited to fill out the questionnaire illustrated in Table 1 by the DEMATEL format from April 2016 to June 2016, but only eleven questionnaires were valid, representing an 84.6% effective response rate. The demographic information about these eleven experts is provided in Table 2, and these experts are from three medical centers, four regional hospitals, and two universities.

Unlike analytic hierarchy process using the consistency ratio to check for the consistency of the decision makers' judgement, the DEMATEL method lacks the consistency ratio evaluation to verify if the comparisons provided by decision makers are consistent [5]. Shieh and Wu [5] proposed an integrated approach of using corrected item-total correlation and split-half methods to evaluate the consistency from the survey data. However, their research conclusion is to identify those experts who have different opinions than the others. In reality, those experts with different opinions might have unique opinions to be taken into account or their opinions which are unreliable should be removed. There is no clear procedure to judge if each decision maker's opinion is valid so far for the DEMATEL method. For instance, the research works published by Professor Gwo-Hshiung Tzeng, an expert in DEMATEL method development and applications, assume the decision makers' opinions are valid without further examining the reliability of the survey results [20–22].

In this study, thirteen experts whose specialties include patient safety culture, medical quality, or human resource management were invited. These experts might have consensus opinions in evaluating the causal relationships among six dimensions. On the other hand, some of them might have their own unique opinions in assessing the causal relationships. In order to incorporate either consensus or individual unique opinions, this study respects each expert's assessment. Therefore, the computations and analyses in the DEMATEL method are based upon the opinions of these eleven experts by following the four major steps depicted in Section 2.

## 4. Results

Eleven 6 × 6 matrices listed in the appendix are eleven experts' opinions on six dimensions. The average matrix **A** based on (1) is as follows:

(3) $$\begin{array}{c} Α=\left[\begin{array}{cccccc} 0 & 2.8333 & 2.7500 & 1.9167 & 2.5000 & 2.6667\\ 2.5000 & 0 & 2.4167 & 2.0000 & 2.3333 & 2.4167\\ 2.6667 & 2.0833 & 0 & 1.9167 & 2.1667 & 2.5833\\ 1.9167 & 2.0833 & 2.3333 & 0 & 2.1667 & 2.4167\\ 2.7500 & 2.2500 & 2.7500 & 1.7500 & 0 & 2.4167\\ 2.5000 & 2.3333 & 2.8333 & 2.0833 & 2.1667 & 0 \end{array}\right]. \end{array}$$

The normalized initial direct-relation matrix **D** based on (2) can be computed below:

(4) $$\begin{array}{c} D=\left[\begin{array}{cccccc} 0 & 0.2237 & 0.2171 & 0.1513 & 0.1974 & 0.2105\\ 0.1974 & 0 & 0.1908 & 0.1579 & 0.1842 & 0.1908\\ 0.2105 & 0.1645 & 0 & 0.1513 & 0.1711 & 0.2039\\ 0.1513 & 0.1645 & 0.1842 & 0 & 0.1711 & 0.1908\\ 0.2171 & 0.1776 & 0.2171 & 0.1382 & 0 & 0.1908\\ 0.1974 & 0.1842 & 0.2237 & 0.1645 & 0.1711 & 0 \end{array}\right]. \end{array}$$

Matrix **T** can be computed by **T** = **D**(**I** − **D**)^−1^ and becomes

(5) $$\begin{array}{c} T=\left[\begin{array}{cccccc} 2.4526 & 2.4776 & 2.7235 & 2.0912 & 2.4240 & 2.6139\\ 2.4212 & 2.1137 & 2.5075 & 1.9365 & 2.2308 & 2.4063\\ 2.5040 & 2.3205 & 2.4106 & 1.9922 & 2.2838 & 2.4842\\ 2.2758 & 2.1427 & 2.3838 & 1.7142 & 2.1201 & 2.2924\\ 2.5385 & 2.3570 & 2.6229 & 2.0028 & 2.1672 & 2.5075\\ 2.4795 & 2.3205 & 2.5881 & 1.9938 & 2.2792 & 2.3069 \end{array}\right]. \end{array}$$

**Figure 1:**
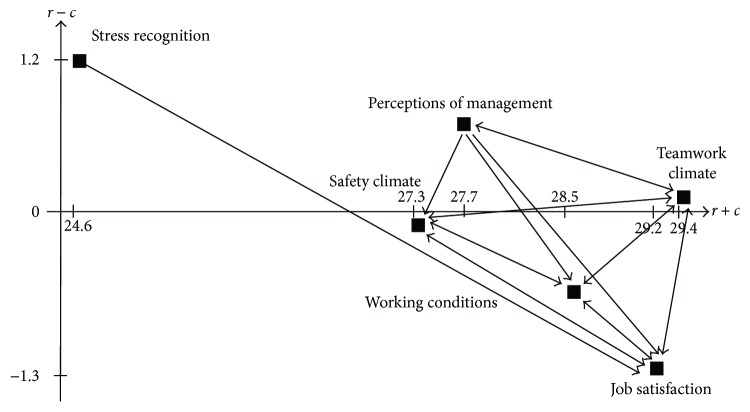
Digraph of six dimensions.

The degree of importance and net effect for each dimension are provided in Table 3, where the importance of six dimensions in terms of (*r* + *c*) values is as follows: teamwork climate > job satisfaction > working conditions > perceptions of management > safety climate > stress recognition. That is, teamwork climate is the most important dimension, whereas stress recognition is the least important dimension. For net effects, teamwork climate, stress recognition, and perceptions of management are net causes with positive (*r* − *c*) values. In contrast, safety climate, job satisfaction, and working conditions are net receivers with negative (*r* − *c*) values. Finally, a threshold value can be set up by computing the average value of all the elements in matrix **T**, and the threshold value is 2.3191. The digraph of six dimensions is shown in Figure 1, and the interaction effects between a pair of dimensions are provided in Table 4.

From Figure 1, teamwork climate, safety climate, and working conditions are mutually influenced. On the other hand, perceptions of management impacts teamwork climate, safety climate, job satisfaction, and working conditions but is influenced by teamwork climate solely. In addition, stress recognition influences job satisfaction but is not affected by the other dimensions. It is interesting to note that job satisfaction is the only dimension that is influenced by all of the dimensions.

Through interactions depicted in Table 4, working conditions and safety climate are affected by perceptions of management, job satisfaction, and teamwork climate along with the mutual affection between these two dimensions. If hospital management is intended to improve working conditions and safety climate, enhancing both dimensions is simply not enough. On the other hand, the improvement on teamwork climate, job satisfaction, and perceptions of management would result in better working conditions and safety climate as well. In contrast, the negative influences from teamwork climate, job satisfaction, and perceptions of management would have influences on working conditions and safety climate negatively.

The focal point is that any improvement initiated by hospital management needs to be planned through causal relationships of dimensions. In this study, teamwork climate is the most essential dimension among six dimensions through causal relationships followed by perceptions of management because these two dimensions are net causes with positive (*r* − *c*) values. It is worth to note that job satisfaction is affected by the other dimensions, which indicate that any influence from teamwork climate, perceptions of management, safety climate, stress recognition, or working conditions would have direct impacts on job satisfaction.

Lee et al. [11] stated that patient safety culture in a healthcare organization might be changed from a longitudinal viewpoint. Their study showed that some critical factors might be shifted from important to unimportant and vice versa. Thus, one might be interested in evaluating whether or not the causal relationships among six dimensions of safety attitudes questionnaire would be changed. In doing so, the results conducted by Lee et al. [2] are compared because their study and our study use the same questionnaire and have the experts from Taiwan. The similarities and differences are summarized below. From the degree of importance, both studies identify that teamwork climate is the most important dimension, while stress recognition is the least important dimension. Moreover, by further considering the causal relationships, both studies agree that teamwork climate is the most essential dimension to the patient safety culture. Teamwork climate has direct influences on four dimensions. That is, the improvement on teamwork climate would bring positive influences on four dimensions.

On the other hand, the priorities of six dimensions based on the degree of importance are slightly different. The priority found by Lee et al. [2] is that teamwork climate > working conditions > job satisfaction > perceptions of management > safety climate > stress recognition, whereas the priority found by this study is that teamwork climate > job satisfaction > working conditions > perceptions of management > safety climate > stress recognition. That is, the importance of working conditions and job satisfaction has been perceived slightly different. The causal and receiving dimensions are somewhat different. Lee et al. [2] summarized that teamwork climate, job satisfaction, perceptions of management, and working conditions are causal dimensions but safety climate and stress recognition are receiving dimensions. In contrast, this study identifies that teamwork climate, stress recognition, and perceptions of management are causal dimensions while safety climate, job satisfaction, and working conditions are receiving dimensions. In summary, both studies found that teamwork climate and perceptions of management are two critical causal dimensions and safety climate is the net cause.

## 5. Conclusions

Improving patient safety culture plays a critical role to enhance patient safety in healthcare organizations relentlessly. Analyzing causal relationships among dimensions enables hospital management to classify dimensions into cause-based and effect-based dimensions. In practice, improvements on effect-based dimensions might receive little effects when a great amount of efforts has been invested. In contrast, the improvement might focus on those causal dimensions in order to receive higher paybacks in reality. This study uses the DEMATEL method based on experts' opinions to identify three causal dimensions including teamwork climate, stress recognition, and perceptions of management. Besides, job satisfaction is the dimension that is influenced by the other dimensions based on a contextual relationship. That is, poor performance from one or more dimensions would deteriorate job satisfaction. In summary, teamwork climate and perceptions of management are the two essential dimensions from causal relationships.

Based on the causal relationships, job satisfaction is related to the other five dimensions. That is, an improvement in one or more dimensions would result in better job satisfaction. Thus, job satisfaction can be viewed as an index to reflect if the other dimension(s) have been enhanced. In addition, teamwork climate influences four dimensions except for stress recognition directly. Perceptions of management also influence four dimensions except for stress recognition. From a practical viewpoint, the more teamwork is exhibited among team members, the more safety of patients is committed [23]. Hospital staffs are more likely to focus on patient safety if more team building activities are developed through formal and/or informal communications. Hospital management needs to put more efforts to encourage and reward cooperation and promote the awareness of patient safety to hospital staffs [23]. When hospital staffs can perceive a positive attitude toward the patient safety from hospital management, a better patient safety atmosphere in a healthcare organization can be established.

## Figures and Tables

**Table 1:** The designed questionnaire.

Dimensions of SAQ	Teamwork climate	Safety climate	Perceptions of management	Job satisfaction	Working conditions	Stress recognition
Teamwork climate	–					
Safety climate		–				
Perceptions of management			–			
Job satisfaction				–		
Working conditions					–	
Stress recognition						–

0: no influence; 1: low influence; 2: medium influence; 3: high influence.

**Table 2:** Demographic information of eleven experts.

	Frequency	Percentage
*Gender*		
Male	5	45.5
Female	6	54.5
*Age*		
21–30 years old	1	9.1
31–40 years old	2	18.2
41–50 years old	7	63.6
51–60 years old	1	9.1
*Education*		
College/university	5	45.5
Master's degree	4	36.4
Doctoral degree	2	18.2
*Working experience*		
5 to 10 years	2	18.2
11–20 years	7	63.6
21 years and above	2	18.2
*Areas of expertise (multiple choice)*		
Patient safety	6	
Medical quality	5	
Human resource management	3	
*Working experience in the area of expertise*		
Less than 1 year	1	9.1
1 to 2 years	0	0
3 to 4 years	1	9.1
5 to 10 years	4	36.4
11–20 years	4	36.4
21 years and above	1	9.1

**Table 3:** The direct and indirect effects of six dimensions.

Dimension	*r + c*	*r – c*
Teamwork climate	29.4544	0.1112
Safety climate	27.3480	−0.1160
Job satisfaction	29.2317	−1.2411
Stress recognition	24.6597	1.1983
Perceptions of management	27.7010	0.6908
Working conditions	28.5792	−0.6432

**Table 4:** Interaction effects between dimensions.

Dimension	Affected dimension(s)
Teamwork climate	Safety climate
	Job satisfaction
	Perceptions of management
	Working conditions

Safety climate	Teamwork climate
	Job satisfaction
	Working conditions

Job satisfaction	Teamwork climate
	Safety climate
	Working conditions

Stress recognition	Job satisfaction

Perceptions of management	Teamwork climate
	Safety climate
	Job satisfaction
	Working conditions

Working conditions	Teamwork climate
	Safety climate
	Job satisfaction

## Appendix

Opinions from eleven experts expressed in terms of eleven 6 × 6 matrices are summarized below.

(A.1) $$\begin{array}{c} {Χ}^{1}=\left[\begin{array}{cccccc} 0 & 3 & 3 & 2 & 2 & 3\\ 3 & 0 & 3 & 3 & 3 & 3\\ 2 & 2 & 0 & 3 & 2 & 3\\ 2 & 3 & 3 & 0 & 3 & 3\\ 2 & 2 & 2 & 1 & 0 & 2\\ 1 & 2 & 3 & 2 & 2 & 0 \end{array}\right],\\ {Χ}^{2}=\left[\begin{array}{cccccc} 0 & 3 & 3 & 0 & 0 & 3\\ 1 & 0 & 3 & 0 & 0 & 3\\ 2 & 0 & 0 & 0 & 0 & 3\\ 0 & 0 & 3 & 0 & 0 & 3\\ 3 & 2 & 3 & 0 & 0 & 3\\ 3 & 1 & 3 & 2 & 0 & 0 \end{array}\right],\\ {Χ}^{3}=\left[\begin{array}{cccccc} 0 & 3 & 3 & 2 & 2 & 3\\ 3 & 0 & 3 & 3 & 3 & 3\\ 3 & 3 & 0 & 3 & 3 & 3\\ 3 & 2 & 3 & 0 & 2 & 3\\ 3 & 2 & 3 & 3 & 0 & 2\\ 3 & 3 & 3 & 2 & 2 & 0 \end{array}\right],\\ {Χ}^{4}=\left[\begin{array}{cccccc} 0 & 3 & 3 & 2 & 3 & 2\\ 3 & 0 & 3 & 2 & 2 & 1\\ 3 & 3 & 0 & 1 & 3 & 3\\ 2 & 2 & 2 & 0 & 2 & 2\\ 3 & 3 & 3 & 0 & 0 & 2\\ 2 & 2 & 3 & 1 & 2 & 0 \end{array}\right],\\ {Χ}^{5}=\left[\begin{array}{cccccc} 0 & 3 & 3 & 3 & 3 & 3\\ 3 & 0 & 3 & 3 & 3 & 3\\ 3 & 3 & 0 & 3 & 2 & 3\\ 2 & 2 & 2 & 0 & 3 & 3\\ 2 & 2 & 2 & 2 & 0 & 3\\ 2 & 3 & 3 & 3 & 2 & 0 \end{array}\right],\\ {Χ}^{6}=\left[\begin{array}{cccccc} 0 & 3 & 3 & 3 & 3 & 3\\ 3 & 0 & 2 & 2 & 2 & 3\\ 3 & 2 & 0 & 2 & 2 & 2\\ 3 & 2 & 2 & 0 & 2 & 2\\ 3 & 2 & 2 & 2 & 0 & 2\\ 3 & 3 & 2 & 2 & 2 & 0 \end{array}\right],\\ {Χ}^{7}=\left[\begin{array}{cccccc} 0 & 2 & 3 & 2 & 3 & 3\\ 1 & 0 & 1 & 1 & 2 & 1\\ 3 & 1 & 0 & 2 & 2 & 2\\ 2 & 1 & 2 & 0 & 2 & 2\\ 3 & 2 & 3 & 2 & 0 & 3\\ 3 & 2 & 3 & 2 & 3 & 0 \end{array}\right],\\ {Χ}^{8}=\left[\begin{array}{cccccc} 0 & 3 & 2 & 1 & 3 & 2\\ 3 & 0 & 3 & 2 & 3 & 2\\ 3 & 3 & 0 & 3 & 1 & 2\\ 2 & 3 & 2 & 0 & 3 & 2\\ 3 & 2 & 3 & 3 & 0 & 2\\ 3 & 2 & 2 & 3 & 3 & 0 \end{array}\right],\\ {Χ}^{9}=\left[\begin{array}{cccccc} 0 & 2 & 2 & 2 & 3 & 1\\ 3 & 0 & 2 & 1 & 3 & 2\\ 3 & 3 & 0 & 2 & 3 & 2\\ 1 & 3 & 3 & 0 & 3 & 2\\ 3 & 3 & 3 & 2 & 0 & 3\\ 3 & 3 & 3 & 2 & 3 & 0 \end{array}\right],\\ {Χ}^{10}=\left[\begin{array}{cccccc} 0 & 3 & 2 & 3 & 3 & 3\\ 3 & 0 & 3 & 2 & 3 & 3\\ 3 & 3 & 0 & 2 & 3 & 3\\ 3 & 3 & 3 & 0 & 3 & 3\\ 3 & 2 & 3 & 3 & 0 & 2\\ 3 & 3 & 3 & 3 & 2 & 0 \end{array}\right],\\ {Χ}^{11}=\left[\begin{array}{cccccc} 0 & 3 & 3 & 1 & 3 & 3\\ 1 & 0 & 1 & 2 & 1 & 2\\ 3 & 1 & 0 & 1 & 3 & 3\\ 1 & 1 & 1 & 0 & 1 & 1\\ 3 & 3 & 3 & 1 & 0 & 3\\ 1 & 1 & 3 & 1 & 3 & 0 \end{array}\right]. \end{array}$$
